# Genetic and epidemiological insights into the emergence of peste des petits ruminants virus (PPRV) across Asia and Africa

**DOI:** 10.1038/srep07040

**Published:** 2014-11-13

**Authors:** Abinash Padhi, Li Ma

**Affiliations:** 1 Department of Animal and Avian Sciences, University of Maryland, College Park, MD-20742, USA

## Abstract

Small ruminants are important components in the livelihood of millions of households in many parts of the world. The spread of the highly contagious peste des petits ruminants (PPR) disease, which is caused by an RNA virus, PPRV, across Asia and Africa remains a major concern. The present study explored the evolutionary and epidemiological dynamics of PPRV through the analyses of partial N-gene and F-gene sequences of the virus. All the four previously described PPRV lineages (I-IV) diverged from their common ancestor during the late-19^th^ to early-20^th^ century. Among the four lineages, PPRV-IV showed pronounced genetic structuring across the region; however, haplotype sharing among the geographic regions, together with the presence of multiple genetic clusters within a country, indicates the possibility of frequent mobility of the diseased individuals across the region. The gradual decline in the effective number of infections suggests a limited genetic variation, which could be attributed to the effective vaccination that has been practiced since 1990s. However, the movement of infected animals across the region likely contributes to the spread of PPRV-IV. No evidence of positive selection was identified from this study.

Recognizing the importance of small ruminants (i.e., sheep and goat) in food security, socio-economic growth, and the livelihood of millions of households predominantly in Africa and Asia as well as in many other parts of the world, the Food and Agriculture Organization (FAO) and World Organization for Animal Health (OIE) have prioritized for complete eradication of the devastating peste des petits ruminants (PPR) disease that causes a very high mortality among small ruminants across Asia and Africa^1^,^2^,^3^,^4^,^5^. This highly contagious viral disease is caused by a non-segmented negative-strand RNA virus, peste des petits ruminants virus (PPRV)^1^,^4^,^5^,^6^,^7^,^8^. The continuing spread of this devastating disease across Asia and Africa appears to be a serious threat to the emerging economy^2^,^4^,^5^. Hence, there is an increasing awareness among researchers and policy makers of the need to fight this livestock-killing disease at a regional and global level^1^,^2^,^3^,^4^,^5^,^9^,^10^.

PPR disease, also known as the goat plague, was first reported in 1942 in Côte d'Ivoire^11^, and has subsequently been reported with high prevalence from African and Asian countries as well as from the European part of Turkey^1^,^4^,^5^,^9^,^12^,^13^,^14^,^15^. Although small ruminants are the principal host of this paramyxovirus, PPRV has been reported to infect several other species, including cattle, buffalo, free-living bharals, camel, Asiatic lion and wild ungulates^9^,^13^,^16^, thus indicating a successful adaptation to a wide range of host species inhabiting different agro-eco-climatic zones. The PPRV genome is approximately 16 kb in length and encodes six structural proteins: the nucleocapsid (N), phosphoprotein (P), matrix (M), fusion (F), hemagglutinin (H) and polymerase (L) proteins, in the order of 3′-N-P-M-F-H-L-5′, and two non-structural proteins, C and V^1^,^5^. Based on the partial nucleotide sequence data of the N^17^ and F^18^ genes, PPRV isolates can be classified into four lineages: I, II, III and IV. Previous reports suggested an extent of geographic specificity of these four lineages. For instance, while lineages I and II, respectively are restricted to western and central Africa, lineage III is restricted to eastern Africa and the Arabian Peninsula^1^,^5^,^9^. Interestingly, lineage IV, which is also referred to as the Asian lineage, has a wide geographic coverage ranging from Southeast Asia to the Middle East, and has more recently expanded into northern Africa^5^. Lineage IV has not only the predominant distribution across the region but also is gradually replacing lineage III in some parts of Africa^5^,^19^,^20^. Although the reason why lineage IV is more widespread than other lineages is unknown, one might speculate that lineage IV may have a selective advantage over other lineages. Under such circumstances, some amino acid residues in the PPRV-IV proteins are expected to evolve adaptively.

Despite the fact that live attenuated vaccines have been widely used to protect small ruminants against circulating PPRV^1^,^3^,^7^, the continuous spread of PPR disease indicates two possible hypotheses: (1) emergence of new PPRV strains with new genetic makeups and greater fitness in the face of vaccine-elicited protection (e.g., like other RNA viruses^21^,^22^); and (2) lapses in regulatory control that ultimately lead to movement of diseased/infected individuals across the region/state/country without proper monitoring and surveillance. Either of these two hypotheses can result in the spread of this highly contagious disease across a wide geographic range. If the first hypothesis holds with an emergence of new PPRV strains that is vaccine-driven or due to antigenic drift (e.g., Mumford et al.^23^), we expect to observe an increase in viral genetic diversity over time as well as footprints of positive selection in viral genes and proteins that play a crucial role in the successful adaptation to new environments, hosts and/or geographic regions. Thus, the central objective of this study is to test the two hypotheses through assessing whether past vaccination efforts have any effect on the viral genetic diversity and whether any of the protein-coding genes in the PPRV genome exhibit adaptive evolutionary changes.

Given the ongoing risk posed by the PPRV, particularly the rapid spread of the PPRV-IV lineage across Asia and Africa, it is crucial to determine whether past vaccination efforts have had any significant effect on the reduction of PPRV viral diversity over time. By performing a Bayesian coalescent analysis^24^,^25^,^26^ using serially sampled partial N- and F-gene sequence data and by performing site-specific maximum likelihood (ML) based selection analyses for each protein-coding gene of PPRV^27^,^28^, we report the evolutionary rate and date of emergence of the currently circulating PPRV, the patterns of selection pressures as well as gain important insights into the epidemiology of this highly contagious paramyxovirus.

## Results

### Dating the emergence of PPR viral lineages

**Figure 1:**
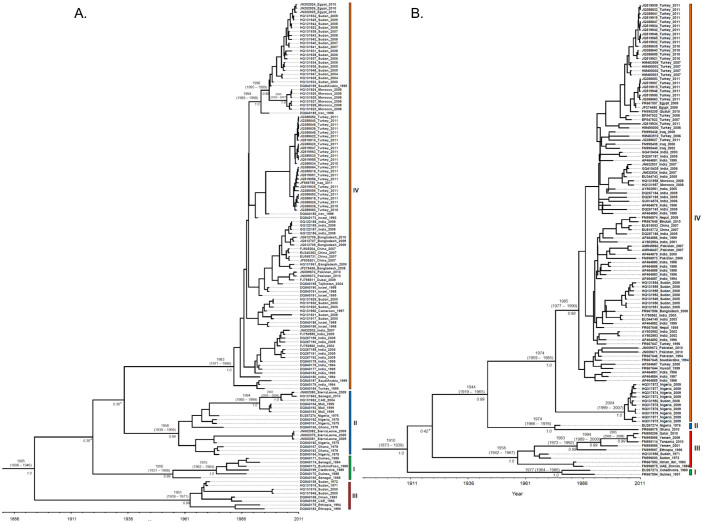
Maximum clade credibility (MCC) trees from the Bayesian analysis of the PPRV partial (A) nucleocapsid and (B) fusion gene sequences. The posterior probabilities and TMRCAs of the branches are depicted. Nucleotide accession number, country of origin, and sampling year of each isolate is shown. Major nodes with weak posterior probability support (< 0.95) are indicated in asterisks (*).

To infer the substitution rate and the time to the most recent common ancestors (TMRCAs) of PPRV lineages, we performed in-depth analyses using a Bayesian-based coalescent approach (Fig. 1A & B; Table 1). The Bayes factor (BF) statistic was used to select the best-fit clock model for the respective data sets. The BF is the difference in the marginal log likelihoods between two nested models: a model with the lowest marginal likelihood score (null model) and the other model with the highest marginal likelihood score (alternative model)^29^,^30^. For both N- and F- genes that comprised of all the four lineages and for the lineage IV of N-gene, the statistic of 2ln(BF) is greater than 20, thus providing strong evidence for a relaxed clock model with uncorrelated lognormal (UCLN) as the best-fit model (Table 1). The inferred substitution rates and TMRCAs for the respective data sets estimated under the best-fitting relaxed model that has the highest marginal log likelihood are shown in Table 1 and the inferred Maximum Clade Credibility (MCC) trees are shown in Figure 1. The MCC trees inferred from the partial N and F gene sequence data revealed the date of emergence of respective lineages of PPRV (Fig. 1A & B; Table 1). Both genes support the existence of four distinct lineages of PPRV (lineages, I-IV) with strong posterior probability support (≥ 0.99). Although both trees showed lineage IV and II clustered together, this inter-lineage clustering was weakly supported in the N-gene tree with posterior probability < 0.95 (Fig. 1A). Nevertheless, both genes showed that all the four lineages of PPRV diverged from a common ancestor. Subtle differences in the mean substitution rates of N- and F- genes of PPRV were observed (Table 1). The lower 95% Highest Posterior Density (HPD) value of Coefficient of Variation (CoV) was close to zero for the F gene, indicating no significant variations in evolutionary rate across the lineages (Table 1). In contrast, the higher CoV observed for the N gene and the 95% HPD not encompassing zero (Table 1) indicate large-scale variation in substitution rates across the lineages. The mean substitution rate for the N gene is higher than that of the F gene. Despite the subtle differences in substitution rates, both genes are consistent with a mean TMRCA of PPRV being around the late-1800s to the early-1900s (Table 1). Both genes also showed consistency in the time of divergence within respective lineages, which was estimated to have occurred between 1931 and 1990 (Fig. 1A & B). Separate analyses for lineage IV also showed that the substitution rate for the N gene is relatively higher than that of the F gene (Table 1). However, the TMRCA estimates from both genes indicate that the diversification within lineage IV was likely to have begun around early-to mid-1980s (Table 1).

### Geographical spread of PPRV –IV

**Figure 2:**
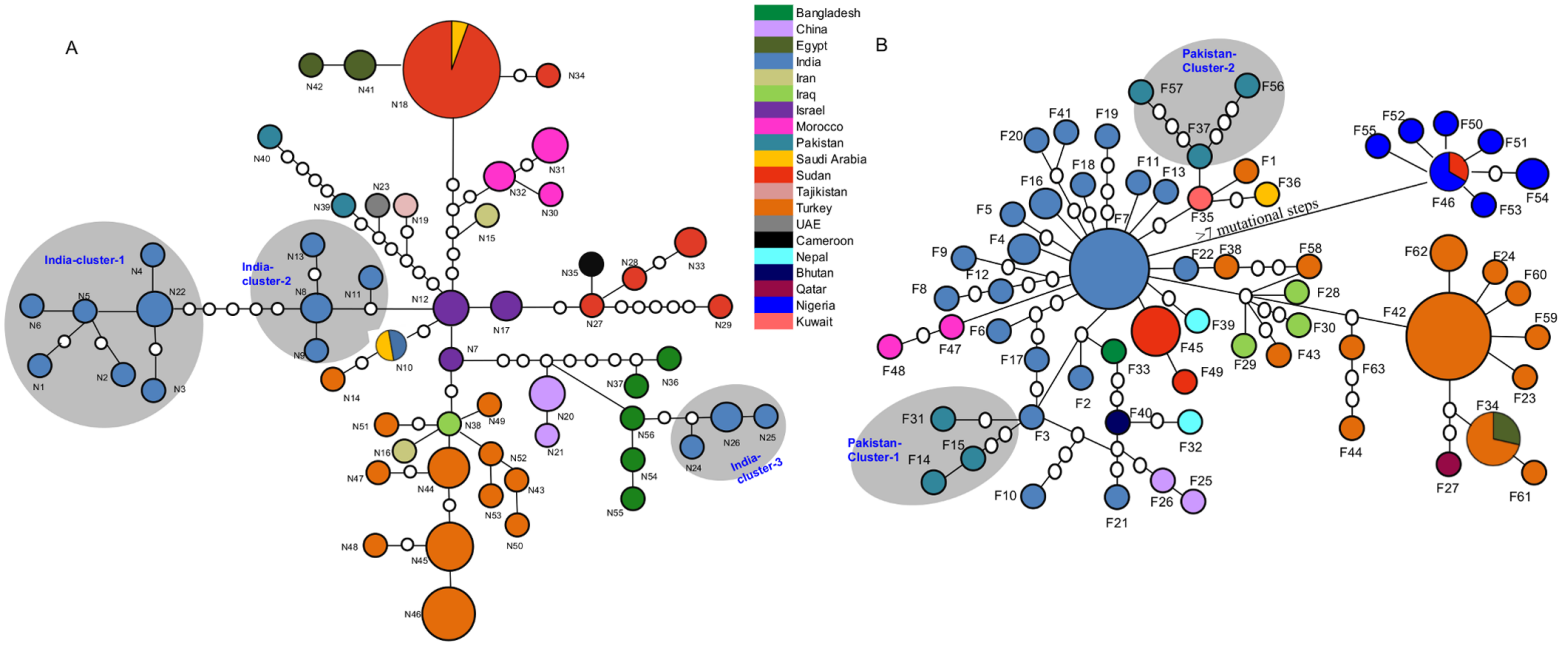
Parsimony networks based on the partial (A) N and (B) F genes of the PPRV isolates belong to lineage IV. Each colored circle represents a unique gene sequence (haplotype), and the size of the circle is proportional to the number of isolates of that haplotype. Country of origin is indicated by unique color. Each empty circle between two adjacent colored circles represents the hypothetical mutational step not observed in the sample. All the isolates were connected at 95% confidence limit (connection limit = 7 mutations). The existence of multiple clusters in India and Pakistan are highlighted in N-and F genes, respectively.

To assess whether each unique sequence (haplotype) of N-and F gene is specific to any geographic location or shared across the wide geographic range, we performed network analyses (Fig. 2A & B). From the 101 N-gene sequences and the 103 F-gene sequences, we obtained 56 (N1-N56) and 63 (F1-F63) haplotypes, respectively. In both genes, while most of the haplotypes showed geographic specificity, haplotypes N18, N10 (Fig. 2A) and haplotypes F46 and F34 (Fig. 2B) are shared among multiple countries. Although both genes showed an extent of geographic clustering of PPRV-IV (Fig. 2A & B), the clustering is more pronounced in the N-gene (Fig. 2A). In the N-gene network, PPRV-IV isolates from India, Bangladesh, Sudan, and Turkey showed an existence of multiple clusters (Fig. 2A). Similarly, in the F-gene network, PPRV-IV isolates from Pakistan, India, Nepal, Sudan, and Turkey also showed the existence of multiple clusters (Fig. 2B). The presence of multiple clusters within a country indicates either multiple waves of introduction of diseased animals from the neighboring countries or the recovery of viral populations from multiple bottlenecks. However, the closer association of a cluster with isolates from another country than with the clusters from the same country indicates that multiple waves of introduction of PPRV from different countries are more likely. For instance, in the N-gene network, the Indian isolates are more closely related with the isolates from Bangladesh in one cluster, whereas isolates from India and Israel are more closely related in another cluster (Fig. 2A). Similar patterns were also observed among the isolates from Pakistan, India, Turkey and Sudan in the F-gene network. Based on the results of the networks, it is likely that multiple haplotypes in individual countries and multiple countries connected by a single haplotype, supporting a relatively important trafficking of infected animals in Asia, Middle-East and more recently Africa. Due to the limited sample size from the respective countries, it was difficult to infer detailed migration and/or phylogeographic patterns. However, future studies, with larger sample size from respective countries, can be carried out for an in-depth understanding of the phylogeographic patterns of the PPRV-IV.

### Estimating the changing genetic diversity of PPRV-IV

**Figure 3:**
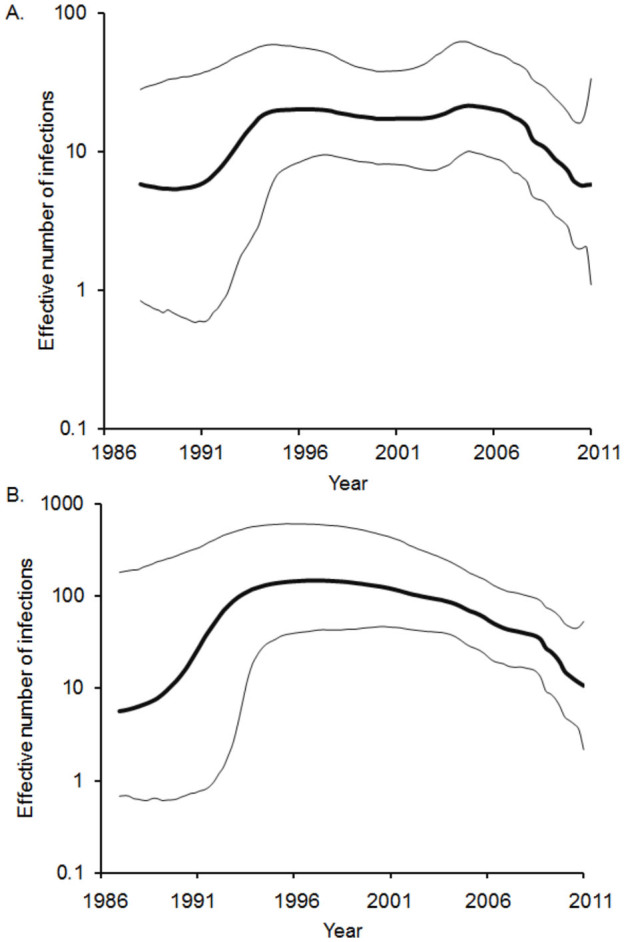
Bayesian skyline plots (BSP) depicting population dynamics of Asian lineage (lineage IV) of PPRV. BSPs inferred from partial (**A**) N gene and (**B**) F gene depicting changing levels of genetic diversity (N_e_τ) of the PPRV-IV sampled between 1987 and 2011. The bold line represents the median estimate; the thin gray lines give the 95% HPD interval of the estimates.

The demographic history of the PPRV-IV was inferred using the Bayesian skyline plot (BSP) (Fig. 3). The effective number of infections estimated from the partial N and F genes showed consistent patterns (Fig. 3). The BSP showed three phases: a sudden increase in viral diversity during the year of 1987, followed by a stable phase until mid- to late-1990s, then followed by a gradual decline thereafter (Fig. 3). The sudden increase in viral diversity during 1987 could be associated with increases in host populations. The estimated genetic diversities for both genes are within the range of 10 to 100, which are consistent with the estimates reported for other paramyxoviruses^31^.

### Measuring selection pressure

Our analysis of selection pressure has revealed that the overall rate of nonsynonymous (dN) to synonymous (dS) substitution (ω = dN/dS) for each gene is less than 1 (Table 2), thus indicating purifying selection is the dominant force in the evolution of PPRV-IV. Although the overall ω indicated evidence of purifying selection, it is still difficult to infer whether any individual codons have evolved under positive selection. If a vast majority of codons have evolved under purifying selection and few codons were subjected to positive selection, the overall ω is expected to be less than 1, and the codons that have evolved under positive selection are likely to be undetected^28^. Considering this fact, we performed codon-specific substitution analyses that accounted for individual codon sites separately. Codon specific-analyses have revealed that, except the M protein, none of the genes showed evidence of positive selection (Table 2), thus indicating the mutations in the respective protein-coding genes are unlikely to have evolved adaptively. The biological significance of the single amino acid that was positively selected in the M protein is not known. Although the present analyses demonstrated the dominant role of purifying selection, sample size could possibly be a limiting factor and therefore, detection of positively selected sites from more samples representing different geographic regions and different hosts cannot be ruled out.

## Discussion

The rapid spread of the highly contagious small ruminants-borne PPR viral disease across the Asian and African continents, which is costing the livelihood of millions of the poorest of the poor's living in the region, warrants urgent attention from the international community^2^,^4^. However, to design effective control measures, an in-depth understanding of the emergence of new viral strains and determining when, where, and how these infectious viral pathogens cause the spread of infectious diseases is crucial^32^. Implementation of phylogenetic approaches to viral DNA sequence data is therefore imperative to elucidate the genetic relationships among different viral isolates sampled from different geographic and/or agro-eco climatic zones to provide important insights into their transmission and epidemiological dynamics. In the present study, we explored the evolutionary and epidemiological dynamics of PPRV through the analyses of the partial N-gene and F-gene sequences.

Previous epidemiological studies reported that PPR was first described in West Africa^11^, and subsequently, the disease was observed in other parts of Africa, followed by in the Middle East in 1983^1^. Although these observations indicated the emergence of PPRV in 1940s, it is very difficult to predict the timing of the emergence of each lineage of PPRV from these previous epidemiological studies. Based on the Bayesian estimates, the currently circulating PPRV is estimated to have originated during the late 19^th^-to early 20^th^ century, and subsequently variants of lineages, I, II, III and IV began diverging during the early- to late-1900s. Consistently, previous studies reported that the TMRCA of the currently circulating measles viruses (MeV), a closely related virus to PPRV, was estimated to be within the last century (around 1889 to 1944)^31^,^33^. The divergence between MeV and RPV (rinderpest virus) was estimated have occurred around the 11^th^ to 12^th^ centuries; however, the effective population size at that time was sufficient for maintaining MeV^33^. Noting that estimated TMRCAs from sampled viral sequences can be very different from the time of origin indicated by historical data due to many reasons, notably, the effects of purifying selection^31^,^34^, the age of PPRV is probably older than what we estimated. Previous studies have demonstrated that branch lengths, whose accuracies are crucial in estimating the rates and TMRCAs, in viral phylogenetic trees, can potentially be underestimated due to purifying selection^34^. Therefore, estimation of intra and/or inter-species (for instance, PPRV, MeV and RPV) from the recently sampled viral pathogens may bias the TMRCA estimates^34^. Nevertheless, taking into account the codon-based substitution models, robust statistical approaches must be developed in order to have in-depth understanding of the ancient origin of the RNA viruses^34^.

Although the underlying genetic mechanisms that caused the evolution and emergence of these four lineages of PPRV are unclear, intuitively, the restricted distributions of lineages, I, II and III indicate that these lineages might have evolved through multiple founder events from a common ancestral population of PPRV in West Africa. The inadvertent introduction of the variants from one of these lineages in the Middle East, possibly through trade, might be a likely cause of the emergence of lineage IV through founder effect and subsequently spread across Asia and a recent re-introduction in Africa. Neutral theory of molecular evolution predicts that the effect of random genetic drift is likely to have a significant effect on small populations^35^, thus genetic accumulation could be extremely rapid in the small colonized viral populations that are founded by a few viral isolates^32^,^36^. Therefore, a founder effect could be a possible explanation for the emergence of these four lineages of PPRV at different time points.

The substitution rates inferred for PPRV are in the range of 10^−3^ to 10^−4^ subs/site/year, and are compatible with the estimates of other RNA viruses, including the paramyxoviruses (e.g.,^31^,^37^,^38^). Such high substitution rates are typical characteristics of RNA viruses^39^; therefore, it does not necessarily mean high levels of antigenic variation^31^,^37^,^39^. The subtle differences in mutation rates among the genes could be associated with the function of the respective genes^3^ and level of selection pressures. The relatively higher mutation rate observed for nucleocapsid gene also reflected the pronounced genetic structuring of the PPRV-IV. Thus, in agreement with previous studies (e.g., Diallo et al.^3^), the present study suggested that N-gene could be a suitable marker for phylogenetic/population genetic studies of PPRV.

The Bayesian skyline plots revealed the population dynamics of PPRV-IV. Both N- and F-genes showed similar patterns, thus indicating both genes have evolved under similar selective pressures. The observed mutations accumulated in these genes are likely due to random genetic drift, which is evidenced by the codon-based selection analyses. Interestingly, the estimates of the viral genetic diversity are between 10 and 100, which are consistent with the estimates reported for other paramyxoviruses^31^. However, the estimates are significantly lower than those previously reported in chronic infections such as HIV^40^,^41^,^42^ and hepatitis C^43^,^44^. Such low levels of PPRV-IV diversity indicate that frequent population bottlenecks are likely to purge out the diseased individuals from the populations, and therefore, limiting the genetic variation^31^. The gradual decline in PPRV-IV diversity since late-1990s could be associated with the effective preventive measures, including the mass vaccination programs adapted in different countries. Previous studies reported that the live attenuated vaccine derived from PPRV strain Nigeria 75/1, belonging to lineage II, was developed in 1989^45^ and was successfully used to vaccinate more than 98,000 sheep and goats^1^,^2^. Subsequently, several vaccines, for instance, Arasur/87, Sungri/96, and Coimbatore/97 have been developed and successfully used in India and other countries worldwide (reviewed in^8^). Such effective vaccination campaign since 1990s could partly explain such a reduction in PPRV-IV viral diversity over time. Therefore, the spread of PPRV-IV infection due to the re-emergence of PPRV-IV resulting from the antigenic variation is highly unlikely. Although there is evidence that RNA viruses (e.g.,^46^,^47^) are prone to establish persistent infection through antigenic variation^23^,^46^, the lack of evidence of positive selection pressures in the viral proteins, notably in F and H genes that are crucial for successful viral adaptation in response to the host immune defense, together with the evidence of continual reduction in genetic diversity since the introduction of the mass vaccination program indicates that the observed patterns of diversity of PPR viruses are unlikely to be due to an antigenic drift.

Nevertheless, although the present study showed an overall reduction in PPRV-IV viral diversity, which indicates the efficacy of current vaccines used and/or correlating with generalized use of efficacious vaccines in some countries, the movement of diseased individuals, as revealed by genetic network analyses, could possibly contribute to the spread of the PPRV-IV virus across the Asian and African continents. Therefore, proper regulatory and bio-security measures should be adhered to in order to help control the spread of this disease.

## Methods

### Sequence data

A total of 131 N gene (255-bp) and 116 F gene (320-bp) partial nucleotide sequences representing diverse geographic regions and four lineages of PRRV were retrieved from GenBank^48^. The GenBank accession number and the year of isolation of each sequence are shown in Figure 1A & B. Sequences were aligned using MEGA version 4^49^. Vaccine strains were excluded from the analyses.

### Estimation of evolutionary parameters and population dynamics

We used a Bayesian Markov Chain Monte Carlo (MCMC) approach to estimate the overall substitution rate (measured in substitutions per site per year) for the N and F genes under the strict (constant molecular clock) and relaxed (uncorrelated lognormal, UCLN) molecular clocks under Bayesian skyline coalescent prior^24^,^26^ implemented in BEAST ver. 1.8.^25^. To select the best-fit evolutionary model for Bayesian MCMC inference, we performed a series of model selection analyses using BEAST. Different substitution and molecular clock models were compared by Bayes Factors (BF), which is the difference in the marginal log likelihoods between two nested models^30^. Large BF values, 2ln(BF) > 3 and >20, respectively indicate positive evidence and strong evidence against the null model (i.e., the model with the lowest marginal log likelihood)^29^. To estimate the evolutionary rates and infer the population dynamics, we used all the nucleotide sequences of the N (n = 131) and F (n = 116) genes whose years of isolation were available. The MCMC chains were run for sufficient time to achieve convergence. Phylogenies were evaluated using a chain length of 30 million states under a General Time Reversible (GTR) model with proportion of invariable sites (I) and gamma distribution shape parameter (G). Uncertainty in the data was described by the 95% highest posterior density (HPD) intervals. Multiple chains were run to access the convergence of trees and were checked using Tracer ver. 1.5 (available at: http://beast.bio.ed.ac.uk/Tracer). The inferred trees were visualized using FigTree ver. 1.4.1 (available at: http://tree.bio.ed.ac.uk/software/figtree/). We considered the node that has a posterior probability > 0.95 as with strong nodal support^50^. We utilized the Bayesian skyline plot (BSP)^26^ as a coalescent prior to infer the population history of PPRV-IV. The BSP estimates of effective population size of PPRV are measured in terms of relative genetic diversity. The changing genetic diversity can be quantified as N_e_τ, where N_e_ is the effective number of infections, and τ is the infection-to-infection generation time^31^. Under relaxed molecular clock model (UCLN), large-scale variation of evolutionary rates in respective data sets were evaluated from the coefficient of variation (CoV) statistic, which represents the scaled variance in evolutionary rate among lineages^24^,^29^. The Maximum Clade Credibility (MCC) tree was generated using the TreeAnnotator software program implemented in the BEAST package.

### Parsimony network

To know how the PPRV-IV isolates from different geographic regions are genetically connected, we performed parsimony network analyses using the viral DNA sequence data. We used partial nucleotide sequence data of the N and F genes to reconstruct the genealogical networks for the respective genes. The network was constructed at a 95% connection limit using TCS 1.21^51^.

### Test for positive selection

Utilizing site-specific codon substitution models^28^, we determined whether any of the amino acid residues in the six protein-coding genes (i.e., N, P, M, F, H, and L) of PPRV-IV are under positive selection. The complete nucleotide sequence data of the respective protein-coding genes were retrieved from GenBank and were used for selection analyses. Prior to selection analyses, a recombination detection program (RDP) implemented in the RDP ver 4.22 software package^52^ was used to determine if any of the genes showed evidence of recombination. The analyses revealed no evidence of recombination, thus allowing us to test for positive selection. We performed ML-based selection analysis to determine whether any of the genes are evolving under positive selection. Several codon-specific models (M7, M8 and M8a) implemented in the codeml program of PAML^27^ were used to determine which residues are evolving under positive selection. Site models allow the rate of nonsynonymous (dN) to the synonymous (dS) ratio (dN/dS = ω) to vary among residues. The input trees for selection analyses were reconstructed using the PhyML program^53^. The likelihood ratio test (LRT) was used to compare the M7 and M8a models that assume no positive selection (ω < 1) with the M8 model that assumes positive selection (ω > 1). Sites with Bayes Empirical Bayes (BEB) posterior probability ≥ 0.95 were considered to evolve under strong positive selection. Positively selected sites were also detected using the Fast Unbiased Bayesian Approximation (FUBAR) method^54^ via the Datamonkey website^55^. A posterior probability greater than 0.95 was used as the threshold for strong evidence of selection in FUBAR.

## Author Contributions

Conceived and designed the experiments: **A.P., L.M**. Performed the experiments: **A.P**. Analyzed the data: **A.P**. Contributed reagents/materials: **L.M**. Wrote the paper: **A.P., L.M**.

## Supplementary Material

Supplementary Information PPRV Nucleocapsid and Fusion gene sequence information

## Figures and Tables

**Table 1:** Bayesian estimates of substitution rates (×10^−3^ nucleotide substitutions per site per year) and TMRCAs (in year) inferred from partial N and F genes. Best-fitting clock models and the corresponding parameter estimates are in bold

Gene	Group	n	Nucleotide substitution model	Clock model	Marginal log likelihood	2ln(BF)	Mean rate in ×10^−3^ subs/site/yr (95% HPD)	TMRCA in year (95% HPD)	Relaxed clock coefficient of variation (95% HPD)
Nucleocapsid	All	131	GTR	Strict	−2027.339		1.05 (0.80–1.31)	1887 (1852–1887)	NA
				Relax	−1989.106	76.466	1.22 (0.83–1.65)	1898 (1818–1944)	*1.08 (0.60*–*1.70)*
			GTR+G	Strict	−2006.608		1.12 (0.83–1.43)	1880 (1838–1912)	NA
				Relax	−1965.744	81.728	1.31 (0.86–1.84)	1893 (1798–1947)	*1.15 (0.65*–*1.79)*
			**GTR+I+G**	Strict	−2006.666		1.11 (0.82–1.44)	1878 (1836–1911)	NA
				**Relax**	−**1965.227**	**82.878**	**1.34 (0.88**–**1.88)**	**1895 (1808**–**1946)**	***1.16 (0.67***–***1.82)***
	Lineage IV	100	GTR	Strict	−1182.504		1.80 (1.31–2.33)	1988 (1984–1992)	NA
				Relax	−1166.582	31.844	1.90 (1.36–2.50)	1988 (1981–1992)	*0.99 (0.32*–*1.78)*
			**GTR+G**	Strict	−1180.553		1.88 (1.33–2.52)	1989 (1984–1992)	NA
				**Relax**	−**1164.267**	**32.572**	**1.96 (1.37**–**2.63)**	**1988 (1981**–**1992)**	***1.05 (0.35***–***1.81)***
			GTR+I+G	Strict	−1181.018		1.89 (1.35–2.53)	1988 (1984–1992)	NA
				Relax	−1165.791	30.454	1.95 (1.34–2.62)	1987 (1981–1992)	0.99 (0.32–1.83)
Fusion protein	All	116	GTR	Strict	−1861.382	1.922	0.68 (0.51–0.87)	1912 (1883–1938)	NA
				Relax	−1862.343		0.69 (0.50–0.86)	1911 (1876–1939)	0.14 (0.0006–0.43)
			**GTR+G**	Strict	−1858.701		0.70 (0.50–0.91)	1911 (1879–1937)	NA
				**Relax**	−**1855.779**	**5.844**	**0.69 (0.50**–**0.91)**	**1910 (1873**–**1939)**	**0.16 (0.0017**–**0.47)**
			GTR+I+G	Strict	−1859.83		0.69 (0.51–0.89)	1911 (1879–1934)	NA
				Relax	−1856.994	5.672	0.69 (0.50–0.90)	1910 (1873–1939)	0.16 (0.0018–0.48)
	Lineage IV	103	**GTR**	Strict	−1410.938		0.78 (0.50–1.1)	1987 (1970–1992)	NA
				**Relax**	−**1403.387**	**15.102**	**0.78 (0.49**–**1.1)**	**1987 (1976**–**1992)**	**0.55 (0.00005**–**1.0)**
			GTR+G	Strict	−1416.085		0.78 (0.49–1.1)	1986 (1968–1992)	NA
				Relax	−1406.868	18.434	0.78 (0.49–1.1)	1987 (1977–1992)	0.55 (0.0002–0.99)
			GTR+I+G	Strict	−1414.696		0.79 (0.50–1.12)	1986 (1971–1992)	NA
				Relax	−1408.031	13.33	0.81 (0.52–1.16)	1987 (1979–1992)	0.56 (0.001–1.01)

n: Number of sequences; BF: Bayes Factor; TMRCA: Time to the most recent common ancestor; HPD: Highest Posterior Density; CoV: Coefficient of Variation. Cases in which the coefficient of variation (CoV) does not encompass zero are in italic.

**Table 2:** The overall rate of nonsynonymous (dN) to synonymous (dS) substitutions and sites under positive selection in the respective protein-coding genes

			PAML					FUBAR
			M7 vs M8		M8 vs M8a			
Gene	n	dN/dS (95% CI)	LRT *(2Δl)*	*p-value*	LRT *(2Δl)*	*p-value*	Positively selected sites	Positively selected sites
N	15	0.19 (0.13–0.28)	0.053	0.97	0.037	0.85	None	None
P	6	0.49 (0.34–0.68)	1.99	0.37	1.88	0.17	None	None
**M**	13	**0.27 (0.18**–**0.39)**	**10.9**	**0.004**	**10.9**	**0.001**	331, **335**	**335**
F	19	0.17 (0.12–0.24)	0	1	0	1	None	None
H	16	0.20 (0.15–0.26)	0	1	0	1	None	None
L	8	0.16 (0.13–0.20)	0	1	0	1	None	None

n:Number of sequences; LRT: likelihood ratio test statistic for null model versus alternative model; 2*Δl*: Differences in the likelihood scores of null and alternative models; Null models (Neutral model): M7 and M8a; Alternative model (Selection model): M8; Degrees of freedom for M7-M8 and M8-M8a are 2 and 1, respectively. Sites with *p* < 0.05 and posterior probability > 0.95 for PAML and FUBAR respectively are considered to be under positive selection. Sites that are detected to be under positive selection by both methods are in bold.
