# Supplementary material for: Genetic and epidemiological insights into the emergence of peste des petits ruminants virus (PPRV) across Asia and Africa
Source: Sci Rep. 2014 Nov 13;4:7040. doi: 10.1038/srep07040 (PMC4229660; doi:10.1038/srep07040)
Supplement: Supplementary Information — PPRV Nucleocapsid and Fusion gene sequence information [file srep07040-s1.pdf]

Title: Genetic and epidemiological insights into the emergence of peste des petits ruminants virus (PPRV) across Asia and Africa

Authors: Abinash Padhi, Li Ma

PPRV Nucleocapsid gene sequence information.

GenBank Nucleotide accession number	Country of origin	Year of isolation
DQ840165	Senegal	1968
DQ840158	Sudan	1972
DQ840161	Nigeria	1975
DQ840162	Nigeria	1975
DQ840160	Nigeria	1975
DQ840163	Ghana	1976
DQ840164	Nigeria	1976
DQ840167	Ghana	1978
DQ840166	Ghana	1978
DQ840168	Oman	1983
DQ840169	UAE	1986
DQ840172	BurkinaFaso	1988
DQ840170	Guinea	1988
DQ840199	CtedIvoire	1989
DQ840171	Guinea	1989
DQ840173	Israel	1993
DQ840174	Senegal	1994
DQ840175	Ethiopia	1994
DQ840176	India	1994
DQ840179	India	1994
DQ840180	India	1994
DQ840177	India	1995
DQ840178	India	1995
DQ840182	India	1995
DQ840181	Israel	1995
DQ840183	Ethiopia	1996
DQ840184	Turkey	1996
HQ131960	Cameroon	1997
DQ840185	Iran	1998
DQ840186	Iran	1998

DQ840191	Israel	1998
DQ840188	Israel	1998
DQ840189	Israel	1998
DQ840190	Israel	1998
DQ840192	Mali	1999
DQ840195	SaudiArabia	1999
DQ840197	SaudiArabia	1999
DQ840198	Tajikistan	2004
HQ131962	CAR	2004
DQ267188	India	2005
DQ267191	India	2005
DQ267192	India	2005
DQ267189	India	2005
DQ267190	India	2005
EU068731	China	2007
EU340363	China	2007
HQ131961	Bangladesh	2009
HQ131963	Senegal	2010
HQ131933	Sudan	2000
HQ131935	Sudan	2004
HQ131947	Sudan	2004
HQ131934	Sudan	2005
HQ131936	Sudan	2005
HQ131948	Sudan	2005
HQ131937	Sudan	2006
HQ131938	Sudan	2006
HQ131939	Sudan	2007
HQ131940	Sudan	2007
HQ131941	Sudan	2007
HQ131942	Sudan	2008
HQ131922	Sudan	2008
HQ131943	Sudan	2008
HQ131944	Sudan	2008
HQ131945	Sudan	2009
HQ131932	Sudan	2009
HQ131931	Sudan	2008
HQ131929	Sudan	2000
HQ131930	Sudan	2000
HQ131920	Sudan	2000
HQ131917	Sudan	2000
HQ131921	Sudan	2008

HQ131918	Sudan	1971
HQ131919	Sudan	2000
HQ131946	Sudan	2000
HQ131923	Morocco	2008
HQ131924	Morocco	2008
HQ131925	Morocco	2008
HQ131926	Morocco	2008
HQ131927	Morocco	2008
HQ131928	Morocco	2008
FJ750560	India_2003	2003
FJ750559	India	2005
JQ519963	Turkey	2011
JQ519959	Turkey	2011
JQ519955	Turkey	2011
JQ519951	Turkey	2011
JQ519947	Turkey	2011
JQ519910	Turkey	2011
JQ519935	Turkey	2011
JQ388650	Turkey	2011
JQ388646	Turkey	2011
JQ388639	Turkey	2011
JQ388634	Turkey	2010
JQ388630	Turkey	2011
JQ388626	Turkey	2011
JQ388622	Turkey	2011
JQ388618	Turkey	2011
JQ388664	Turkey	2011
JQ388660	Turkey	2010
JQ388656	Turkey	2011
JQ388652	Turkey	2011
JQ388648	Turkey	2011
JQ388640	Turkey	2011
JQ388636	Turkey	2010
JQ388620	Turkey	2011
JQ388616	Turkey	2011
JF969755	Iraq	2011
JN602079	SierraLeone	2009
JN602080	SierraLeone	2009
JN602081	SierraLeone	2009
JN602082	SierraLeone	2009
DQ840193	Mali	1999

DQ840194	Mali	1999
EU267274	Nigeria	1976
JN202926	Egypt	2010
JN202925	Egypt	2010
JN202924	Egypt	2010
JN632532	India	2007
JF276436	Bangladesh	2008
JN009673	Pakistan	2010
JN009674	Pakistan	2010
FJ795511	Dubai	2009
GQ122187	India	2008
GQ122186	India	2008
GQ122188	India	2008
GQ122189	India	2008
JQ612709	Bangladesh	2009
JQ612706	Bangladesh	2010
JQ612707	Bangladesh	2009
FJ905304	China	2007
JF939201	China	2007

PPRV Fusion gene sequence information.

GenBank Nucleotide accession number	Country of origin	Year of isolation
FJ750562	India	2003
JQ519965	Turkey	2011
JQ519948	Turkey	2011
JQ519942	Turkey	2011
JQ519934	Turkey	2011
JQ519921	Turkey	2010
JQ519907	Turkey	2011
JQ519950	Turkey	2011
JQ519946	Turkey	2011
JQ519939	Turkey	2011
JQ519932	Turkey	2011
JQ519924	Turkey	2011
JQ519919	Turkey	2011
JQ519915	Turkey	2011
JQ388663	Turkey	2011
JQ388659	Turkey	2010
JQ388647	Turkey	2011
JQ388643	Turkey	2010
JQ388635	Turkey	2010
JQ388627	Turkey	2011
JQ388653	Turkey	2011
JQ388641	Turkey	2011
JQ388633	Turkey	2011
AY602984	India	2001
AY602982	India	2002
AY602983	India	2002
AY602981	India	2000
JN632531	India	2007
JN632534	India	2007
JN009671	Pakistan	2010
JN009672	Pakistan	2010
JF274480	Egypt	2009
HQ317878	Nigeria	2009
HQ317876	Nigeria	2009
HQ317874	Nigeria	2009
HQ317872	Nigeria	2009
HQ317879	Nigeria	2009

HQ317877	Nigeria	2009
HQ317875	Nigeria	2009
HQ317873	Nigeria	2009
HQ317871	Nigeria	2009
HQ131959	Sudan	2008
HQ131957	Morocco	2008
HQ131955	Sudan	2008
HQ131953	Sudan	2008
HQ131951	Sudan	2005
HQ131949	Sudan	2005
HQ131958	Morocco	2008
HQ131956	Sudan	1971
HQ131954	Sudan	2009
HQ131952	Sudan	2008
HQ131950	Sudan	2005
FR667648	Nepal	1995
FR667646	Pakistan	1994
FR667644	Kuwait	1999
FR667556	Bangladesh	2000
FR667554	Guinea	1991
FN996974	Nepal	2009
FN996000	Sudan	1972
FN995998	Yemen	2009
FN995439	Iraq	2000
FR667649	Bhutan	2010
FR667647	Turkey	1996
FR667645	SaudiArabia	1994
FR667557	Egypt	2009
FN996973	Pakistan	2009
FN995999	Yemen	2001
FN995997	Ethiopia	1996
FN995440	Iraq	2002
FN995438	Iraq	2000
HM483509	Turkey	2007
HM483510	Turkey	2006
HM490003	Turkey	2007
HM490001	Turkey	2007
HM490002	Turkey	2007
HM490000	Turkey	2006
GU014576	India	2006
EU344740	India	2003

EU344743	India	2005
AF464892	India	1996
AF464890	India	1999
AF464888	India	1998
AF464886	India	1999
AF464884	India	1997
AF464882	India	1999
AF464880	India	1998
AF464878	India	1998
AF464891	India	1998
AF464889	India	1998
AF464887	India	1994
AF464885	India	1996
AF464883	India	1996
AF464881	India	1999
AF464879	India	2000
AF384687	Turkey	2000
GQ410434	India	2003
GQ410435	India	2006
EU816772	China	2007
EU815053	China	2007
AM946407	Pakistan	2007
AM945963	Pakistan	2007
EF547922	Turkey	2006
EF547923	Turkey	2007
DQ267187	India	2005
DQ267185	India	2005
DQ267183	India	2005
DQ267186	India	2005
DQ267184	India	2005
EU267274	Nigeria	1976
EU267273	CotedIvoire	1989
FR668075	Ghana	2010
FR667553	Oman	1983
FN996975	UAE	1986
FN995205	Quatar	2010
FN995114	Tanzania	2010
FN995206	Qatar	2010
